# Analysis of Select Dietary Supplement Products Marketed to Support or Boost the Immune System

**DOI:** 10.1001/jamanetworkopen.2022.26040

**Published:** 2022-08-10

**Authors:** Cindy Crawford, Bharathi Avula, Andrea T. Lindsey, Abraham Walter, Kumar Katragunta, Ikhlas A. Khan, Patricia A. Deuster

**Affiliations:** 1 Consortium for Health and Military Performance, Department of Military and Emergency Medicine, F. Edward Hebert School of Medicine, Uniformed Services University, Bethesda, Maryland; 2 Henry M. Jackson Foundation for the Advancement of Military Medicine, Bethesda, Maryland; 3 National Center for Natural Products Research, School of Pharmacy, University of Mississippi, University

## Abstract

**Importance:**

Cold, flu, and immunity dietary supplement product sales have skyrocketed since the start of the COVID-19 pandemic. Supporting or boosting the immune system has become an important reason for using dietary supplements, and many consumers are purchasing products through online platforms.

**Objectives:**

To examine whether select dietary supplement products advertised as supporting or boosting the immune system are accurately labeled according to the Supplement Facts label of listed ingredients and to qualitatively describe the product labels’ characteristics in terms of claims made.

**Design, Setting, and Participants:**

In this case series, 30 featured immune health dietary supplements were selected and purchased from Amazon.com in May 2021. Product analysis was performed using liquid chromatography–mass spectrometry. The list of ingredients detected through analysis for each product was compared with the ingredients on the product’s Supplement Facts label to determine whether the product’s label was accurate. Claims made on product labels were also evaluated by using the Operation Supplement Safety Scorecard’s set of questions to describe the labels’ characteristics.

**Results:**

A total of 30 select dietary supplement products were evaluated. Thirteen of the 30 products had accurate labels based on the product analysis. Of the 17 products with inaccurate labels, 13 had ingredients listed on the labels that were not detected through analysis, such that their labels were misbranded. Nine products had substances detected that were not claimed on the product labels, some of which may be considered adulterated. Five were misbranded and contained additional components not claimed on the label. No product had third-party certification seals present on the packaging. Ten of the 13 products with accurate labels received a score of 4 or more when applying the Operation Supplement Safety Scorecard, meaning the product was “likely okay/less risky.”

**Conclusions and Relevance:**

In this case series study, most of the products tested had inaccurate labels and claims that were inconsistent with requirements the US Food and Drug Administration has put forward for dietary supplements. Quality control measures seem to be insufficient for most of these select products, and claims made on labels may be misleading consumers who purchase products.

## Key Points

**Question:**

Are select dietary supplement products advertised and sold to support or boost the immune system accurately labeled according to the Supplement Facts listed ingredients on product labels?

**Findings:**

This case series study analyzed 30 dietary supplement products purchased from Amazon.com with claims related to immune health. Seventeen of 30 products had inaccurate labels; 13 were misbranded, and 9 had additional components detected but not claimed on the label.

**Meaning:**

Quality control measures seem insufficient for most select dietary supplement products. The public has a right to know that they are buying what is stated on the label.

## Teaser

This case series study examines the labeling of select dietary supplement products advertised as supporting or boosting the immune system for accurate ingredient labeling and claims made.

## Introduction

Cold, flu, and immunity supplement sales and growth have sky-rocketed since the start of the COVID-19 pandemic. In 2019, the *National Business Journal* reported immunity supplement sales at approximately $3.4 billion US dollars,^1,2^ and by the end of 2020, those numbers increased to almost $6 billion.^3^ Supporting immunity or boosting the immune system has become an important reason for using dietary supplement products even though some claims may be misleading or not scientifically accurate.^1,4,5^ In addition, information regarding whether there are any risks associated with such products and ingredients is lacking.

In 1994, the Dietary Supplement Health and Education Act (DSHEA) was enacted.^6^ This law defined dietary supplements and the regulatory authority over dietary supplements. Unlike drugs, dietary supplements are not approved by the US government for safety or efficacy. In addition, the US Food and Drug Administration (FDA) does not approve their labeling before going to market. However, the FDA has specific regulations that manufacturers are required to follow for product manufacturing and labeling. The manufacturer is responsible for ensuring their product is safe and lawful and that the claims made are truthful, not misleading, and substantiated. The FDA’s role after market is to inspect manufacturing facilities for product quality and labeling, monitor adverse event reports, and take action to remove any adulterated or misbranded products. The Federal Trade Commission, with the FDA, is responsible for stopping any deceptive and unfair practices in commerce, including false and unsubstantiated marketing claims on dietary supplement products. When the DSHEA law was enacted, the market for dietary supplements was approximately $4 billion, with only approximately 4000 products available.^7,8^ At that time, the “World Wide Web” as a public internet service was merely 3 years old, and Amazon would be launching its first online bookstore in 1995.

Today, across the entire dietary supplement market, e-commerce sales and growth have increased exponentially from $5 billion in 2019 to an estimated $8.4 billion in 2020 supplement sales. e-Commerce represents 15.1% of the total supplement market and 77% of supplement sales happen through Amazon.^9^ The growth in e-commerce, sales, and manufacturing of dietary supplements, coupled with the limited resources for regulating dietary supplements, has created a challenge in keeping up with the market.^10^ Adulteration, misbranding, and misleading claims are regularly reported.^11,12,13,14,15,16,17^ Since December 2020, Amazon has been requiring sellers to provide outlined quality control documentation and a certificate of analysis (testing results) for supplements sold on their platform.^18^ These new requirements could help ensure products are less risky and not adulterated. In addition, and because of the frequent use of dietary supplements by US military service members, a US Department of Defense (DOD) Instruction (DODI) on the Use of Dietary Supplements in the DOD became effective in March 2022. This policy was established to provide guidance and ensure safe dietary supplement use, minimize risks to the military of potential life-threatening adverse events that result from dietary supplement use, and prevent potential disciplinary actions from using supplements with ingredients on the DOD list of prohibited substances. The DODI established Operation Supplement Safety (OPSS) as the go-to program for dietary supplements.^19,20^

In this case series study, we analyzed and qualitatively described select dietary supplements advertised and sold by Amazon and marketed to support and boost the immune system. We tested the products to determine whether their product labels were accurate and whether any product was misbranded or adulterated. We then scored the products according to the online OPSS Scorecard^21^ to assess relative risk based solely on label claims.

## Methods

In this case series, 30 products were selected and purchased from Amazon since the introduction of the new requirements by Amazon in December 2020. On the Amazon website, we searched the key word *immune* in “all departments” and then sorted results by “featured.” The first 30 dietary supplement products that appeared as results with 4 or more stars were eligible and selected for analysis in May 2021. We did not seek institutional review board approval because this case series evaluated products not patients. This study followed the reporting guideline for case series.

One sample of each selected product was purchased and sent to the University of Mississippi’s National Center for Natural Product’s Research for product analysis. Liquid chromatography–mass spectrometry was used to determine the quality of the 30 dietary supplement products. Full details are reported elsewhere^15,22,23^ and fully detailed in the eMethods in the Supplement. All the compounds or ingredients listed on the label and hidden compounds were analyzed except for lipids and elements. The list of ingredients detected through analysis for each product was compared with the ingredients on the product’s Supplement Facts label to determine whether the product’s label was accurate.

Product labels were examined before the testing results and evaluated by using the set of questions from the OPSS Scorecard, which was developed as an educational tool.^15,21,23,24,25^ The Scorecard was designed to help consumers learn about the product and quickly assess whether it might be risky based on the Supplement Facts label and label claims. The scoring could then help them make an informed choice before purchasing the dietary supplement. The Scorecard makes the consumer carefully look at the information presented on the label, including claims made, whether products had third-party certification seals, the number of ingredients on the Supplement Facts label, and whether the product contained proprietary blends or complexes. A score of 4 or more indicates the product is “likely okay/less risky” (based solely on label claims), whereas a score less than 4 is considered “no-go/risky”; the educated consumer might want to choose a different product.^21^ All analyses were conducted in Excel (Microsoft Inc).

## Results

### Product Analysis

A total of 30 select dietary supplement products were evaluated. Thirteen of the 30 products had accurate labels based on the product analysis. Of the 17 products with inaccurate labels, 13 had ingredients listed on the labels that were not detected through analysis, such that their labels were misbranded. Ingredients missing from products ranged from 1 to 6 ingredients from any single product. Ingredients labeled but missing from products (misbranded) included mainly plant extracts, such as *Aloe vera* leaf extract, astragalus root extract (*Astragalus membranaceus*), eleuthro (*Eleutherococcus senticosus*) root, garlic bulb extract (*Allium sativum L*.), ginger root extract (*Zingiber officinale L.*), horehound (*Marrubium vulgare*), *Isatis tinctoria* root extract, Japanese catnip (*Schizonepeta tenuifolia*), licorice (*Glycyrrhiza glabra*), and slippery elm bark (*Ulmus rubra* Muhl). Vitamin B_12_ and folate were missing from 2 products. One product had only traces of elderberry detected. These ingredients along with compounds are listed in the eMethods and eTable in the Supplement.

Nine products had substances detected but not claimed on the product label. One ingredient not claimed on the label but found in 3 products marketed as containing elderberry was *Oryza sativa* (black rice seed). The analysis also detected other substances not claimed on the label, and these substances consisted mostly of flavonoids in 2 products. Pantothenic acid was another hidden (not claimed) ingredient detected in 1 product. Polyethylene glycols were detected in 1 product; it can be an excipient, but it was not claimed on the label. One product listed “licorice root powder (deglycerrhizinated)” on the label. Deglycerrhizinated means removal of glycyrrhizin from licorice, but glycyrrhizin was detected. Lastly, berberine derivatives were identified in 1 product without being claimed on the label (Table; eMethods in the Supplement).

**Table.** Product Analysis of 30 Dietary Supplements Marketed for Immune Health

Product No.	No. of ingredients presented on product label	Product label verification accuracy	No. of ingredients not detected/total No. analyzable for verification^a^	Additional or hidden components detected not present on the label
1	2	Accurate^a^	0/0	None
2	1	Accurate^a^	0/0	None
3	6	Not accurate^a^	0/1	Polyethylene glycols, which can be an excipient, but no claim on the label and may be an adulterant
4	4	Not accurate^a^	0/2	Flavonoids, including isorhamnetin, kaempferol, rutin, isoquercetin, and myricetin
5	8	Not accurate^a^	1/6	Black elderberry extract adulterated with *Oryza sativa* (black rice)
6	9	Accurate^a^	0/2	None
7	13	Not accurate^a^	1/9	None
8	5	Accurate^a^	0/3	None
9	18	Not accurate^a^	0/6	Pantothenic acid
10	15	Not accurate^a^	2/8	None
11	10	Not accurate^a^	1/9	None
12	7	Not accurate^a^	1/5	None
13	12	Not accurate^a^	3/11	Licorice root powder (deglycerrhizinated) was listed on label: deglycerrhizinated means removal of glycyrrhizin from licorice but glycyrrhizin was detected (not counted as an adulterant)
14	6	Not accurate^a^	0/3	Flavonoids, including isorhamnetin, kaempferol, rutin, isoquercetin, and myricetin
15	7	Accurate^a^	0/6	None
16	9	Accurate	0/9	None
17	18	Not accurate^a^	3/16	Berberine analogues were identified not on label, which included dihydromethoxyberberine, oxyberberine, berberine carboxylate, and others
18	32	Not accurate^a^	6/24	Elderberry fruit extract adulterated with *Oryza sativa*
19	17	Not accurate^a^	3/12	None
20	8	Accurate^a^	0/6	None
21	9	Not accurate^a^	1/5	None
22	8	Not accurate^a^	1/6	Black elderberry extract adulterated with *Oryza sativa*
23	14^b^	Accurate^a^	0/14	None
24	5	Accurate^a^	0/4	None
25	6	Accurate^a^	0/2	None
26	4	Accurate^a^	0/2	None
27	13	Accurate^a^	0/6	None
28	17	Not accurate^a^	4/12	None
29	2	Accurate	0/2	None
30	27	Not accurate^a^	6/15	None

^a^ The number of analyzable ingredients differs from the number of ingredients presented on the product label because the instrumentation limits for analysis. Elements, including zinc, magnesium, selenium, calcium, chromium, manganese, potassium, and sodium, fat soluble vitamins, including vitamin D_3_, or any pre- or probiotic ingredients could not be analyzed. In addition, complex polysaccharides presented in fungi are difficult to characterize and quantify, such as mushrooms.

^b^ This product listed ingredients in “other ingredients” that should have been listed in the Supplement Facts panel. “Not detected ingredients” does not totally mean not added, but the amount added might be so low that the instrument could not detect and moreover the low-quantity ingredients may not have required biological activities.

### Product Descriptions

The 30 immune health dietary supplement products tested and analyzed had claims related to immune support, immune defense, and bolstering or boosting the immune system. Examples of such claims included “all seasons immune support,” “immune strengthening ingredients,” “a powerhouse immune system booster,” and “bolster up your body’s immune support.” Fifteen products additionally had scientific sounding claims by using terms such as “research based” or “research supported,” “clinically studied,” scientifically proven,” “supported by…gold-standard clinical studies,” and “backed by science.” No product had a third-party certification seal (ie, naming the third-party company), such as BSCG (Banned Substances Control Group), NSF (National Sanitation Foundation) International, Informed Sport, or USP (US Pharmacopeia), presented on the label. Sixteen products had other seals such as “#1 doctor recommended brand,” “third party tested,” “purity and potency,” “stimulant free,” “lab tested verified,” and “quality guaranteed.” The price of these products ranged from $11.93 to $90.48 for an approximate 30-day supply, with the median cost of $25.33 per month.

The total number of ingredients listed on the Supplement Facts labels of these products ranged from a single ingredient in 1 product to up to 32 listed ingredients. Seven of the products had fewer than 6 ingredients. Twelve products listed blends or proprietary complexes as part of their Supplement Facts label. Of the 312 total ingredients listed across these products, ingredients consisted of herbs and other botanicals (143 [45.8%]), vitamins (74 [23.7%]), minerals (59 [18.9%]), amino acids (6 [1.9%]), and other substances, such as enzymes, organ tissue, and metabolites (12 [3.8%]). Prebiotics and probiotics (7 [2.2%]) and various mushroom extracts (11 [3.5%]) were also included in some products. The most frequent ingredients listed within these categories, respectively, across products were echinacea (14 products), elderberry (18 products), vitamin C (24 products), vitamin D (15 products), and zinc (25 products) (Figure). Twenty-four products either listed no percent daily values or had daily values greater than 200% on the Supplement Facts label. Of these 30 featured products on the Amazon website, only 13 (43.3%) received a score of 4 or more when applying the OPSS Scorecard, and 10 of the 13 (76.9%) had accurate labels confirmed through product analysis.

**Figure.**
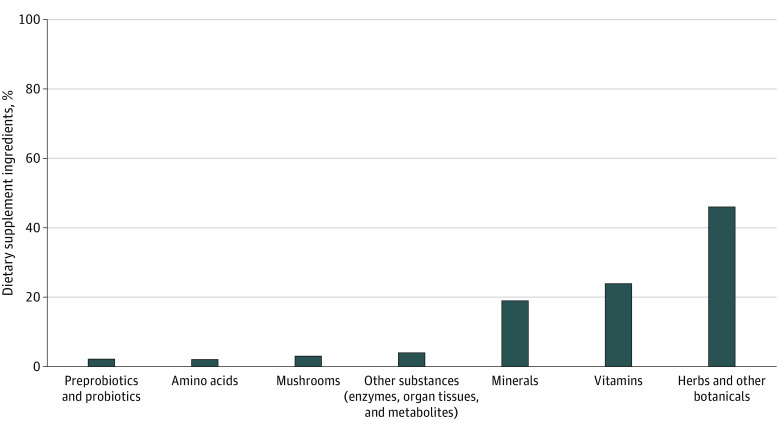
Types of Dietary Supplement Ingredients Advertised on Product Labels Across 30 Dietary Supplements Containing a Total of 312 Ingredients Marketed for Immune Health

## Discussion

Claims made on the labels of most dietary supplement products seem to stretch what would be considered as allowable claims, which can by statute and/or FDA regulations be made for dietary supplements.^26,27^ Some other claims sounded scientific but did not have any peer-reviewed publication cited. Therefore, it is unknown how or whether these claims were substantiated. In a separate effort, we are evaluating the evidence of select dietary supplement ingredients frequently presented in these products to further determine whether claims made are reflective of the existing science concerning their safety and efficacy for otherwise healthy individuals who are looking to support or boost their immune system.

The OPSS program is a DOD-wide educational program established in 2012 to increase awareness within the DOD community about potential health risks and benefits of choosing dietary supplements and to disseminate information on how to choose safe supplements. The OPSS Scorecard^21^ was developed as an educational tool to help consumers make an informed choice. The Scorecard allows the consumer to quickly learn about and screen the safety of a dietary supplement product according to 7 questions. These questions are intended to bring awareness to the consumer and encourage them to carefully examine a product label before making a purchase. A score of less than 4 is classified risky, whereas a score of 4 or more is considered less risky. This educational approach has been useful in helping consumers discern which products may be more safe and less risky.^24,25^ As noted, most products with accurate labels received a score of 4 or more when applying the OPSS Scorecard. Thus, the Scorecard may provide some level of safety to consumers. It is hoped that consumers educated in using the Scorecard would not buy as many of the inaccurately labeled products; however, at the time we purchased these products for analysis, they were listed as the top featured products on the Amazon website.

Dietary supplements, which consumers buy to improve their health, can be costly. The mean price of the 17 products scoring less than 4 was approximately $25 for a 30-day supply, and the mean price for those scoring 4 or more was $31. The public has a right to know that they are buying what is stated on the label when spending money on dietary supplements. This is certainly not always the case, as we found that only 13 of the 30 products were accurate. Purchasing a product with an established third-party certification or verification seal should ensure accuracy in product content. This would certify that the ingredients listed on the label are actually in the container; however, it does not ensure a product’s effectiveness or safety. The new DODI for dietary supplements noted above requires dietary supplement education and prohibits US military service members from taking any product with ingredients on the list of prohibited substances, which should provide some level of risk reduction.^19^ Although we cannot assume any product will confer a benefit, we would certainly not expect any harm; however, there is a risk that misbranded and/or adulterated products could cause harm.

### Limitations

This analysis has some limitations. First, these 30 products are not meant to be representative of all dietary supplement products marketed for immune health and are not representative of all products sold on the Amazon website or any e-commerce market. In addition, the first 30 products that might appear in a search on the Amazon website likely fluctuate on a daily if not hourly basis, depending on industry, keywords, and search engineering. Second, the developed analytical method for product analysis is good for analysis of botanical ingredients, single compounds (synthetic or natural ones), water-soluble vitamins, and amino acids but is not sensitive for analysis of polysaccharides, lipids, enzymes, and proteins. Therefore, elements such as zinc, magnesium, calcium, selenium, probiotic and prebiotic ingredients, mushrooms, and vitamin D_3_ could not be verified through analysis and therefore are not included as ingredients for analysis. We do not know whether these ingredients were present. Third, this analysis was not quantitative in nature; therefore, we cannot comment on whether the amount of each ingredient listed on product labels matched what was detected from analysis.

## Conclusions

This case series analysis suggests that quality control measures have not been sufficient for most immune health dietary supplement products advertised and sold on the Amazon website. Moreover, some claims made on most of these immune support products do not appear consistent with any of the categories of claims defined by FDA regulations. Most products tested had inaccurate labels, and the claims made on those labels may mislead consumers into purchasing products when information on whether they are actually beneficial is limited. Consumers should be aware that these products may potentially not contain what is stated on the label. Continued research and evidence-based educational resources will assist consumers in making informed decisions about dietary supplements.

## Notes

Supplement. **eMethods.** Analytical Methods **eTable.** Phytochemicals Reported for the Botanical Plant Parts Used in the Study

## References

[1] Brush M. Immunity is everything: as the pandemic brings attention to the immune system, many question the attitudes and assumptions about it. Nutr Bus J. 2020:6-9.

[2] Juntti M. The whole pandemic package: immunity is just the start as consumers scoop up supplements for sleep, stress and more. Nutr Bus J. 2020:1-5.

[3] Brush M. Don’t talk about vaccines: Supplement brands wary to wade into the fray, for two big reasons. Nutr Bus J. 2021;(October):1-7.

[4] Hamulka J, Jeruszka-Bielak M, Górnicka M, Drywień ME, Zielinska-Pukos MA. Dietary supplements during covid-19 outbreak: results of Google trends analysis supported by PLIFECOVID-19 online studies. Nutrients. 2020;13(1):E54. doi:10.3390/nu13010054 33375422 PMC7823317

[5] Rachul C, Marcon AR, Collins B, Caulfield T. COVID-19 and ‘immune boosting’ on the internet: a content analysis of Google search results. BMJ Open. 2020;10(10):e040989. doi:10.1136/bmjopen-2020-040989 33109677 PMC7592272

[6] S.784—Dietary Supplement Health and Education Act of 1994. 103rd Congress (1993-1994). Accessed June 10, 2022. https://www.Congress.Gov/bill/103rd-congress/senate-bill/784

[7] Commission on Dietary Supplement Labels. Report of the Commission on Dietary Supplement Labels. Commission on Dietary Supplement Labels; 1997. Accessed June 27, 2022. https://ods.od.nih.gov/pubs/DSHEA1997report.pdf

[8] Institute of Medicine and National Research Council US Committee on the Framework for Evaluating the Safety of Dietary Supplements. *Dietary Supplements: A Framework for Evaluating Safety**.* National Academies Press; 2005. Accessed June 27, 2022. https://www.ncbi.nlm.nih.gov/books/NBK216051/

[9] Brush M. Amazon as crystal ball: can natural brands and retailers use e-commerce to predict trends? Nutr Bus J. 2021:13-15.

[10] Cohen PA. The FDA and adulterated supplements—dereliction of duty. JAMA Netw Open. 2018;1(6):e183329-e183329. doi:10.1001/jamanetworkopen.2018.3329 30646231

[11] Cohen PA, Wang YH, Maller G, DeSouza R, Khan IA. Pharmaceutical quantities of yohimbine found in dietary supplements in the USA. Drug Test Anal. 2016;8(3-4):357-369. doi:10.1002/dta.1849 26391406

[12] Cohen PA, Wen A, Gerona R. Prohibited stimulants in dietary supplements after enforcement action by the US Food and Drug Administration. JAMA Intern Med. 2018;178(12):1721-1723. doi:10.1001/jamainternmed.2018.4846 30422217 PMC6583602

[13] Cohen PA, Zakharevich I, Gerona R. Presence of piracetam in cognitive enhancement dietary supplements. JAMA Intern Med. 2020;180(3):458-459. doi:10.1001/jamainternmed.2019.5507 31764936 PMC6902196

[14] Crawford C, Deuster PA. Be in the know: dietary supplements for cognitive performance. J Spec Oper Med. 2020;20(2):132-135. doi:10.55460/9ANO-BXRD 32573750

[15] Crawford C, Walter AR, Avula B, . Relative safety and quality of various dietary supplement products U.S. Service Members ask about. Clin Toxicol (Phila). 2022;60(6):737-744. doi:10.1080/15563650.2022.2036751 35156875

[16] Tucker J, Fischer T, Upjohn L, Mazzera D, Kumar M. Unapproved pharmaceutical ingredients included in dietary supplements associated with US Food and Drug Administration warnings. JAMA Netw Open. 2018;1(6):e183337. doi:10.1001/jamanetworkopen.2018.3337 30646238 PMC6324457

[17] White CM. Continued risk of dietary supplements adulterated with approved and unapproved drugs: Assessment of the us food and drug administration’s tainted supplements database 2007 through 2021. J Clin Pharmacol. Published online March 14, 2022. doi:10.1002/jcph.2046 35285963

[18] Amazon’s Dietary Supplement Policy. Accessed March 4, 2021. https://sellercentral.amazon.com/gp/help/external/55N3JF2WQS7RVNE

[19] US Department of Defense. Use of dietary supplements in the DoD. Accessed June 10, 2022. https://www.opss.org/sites/default/files/2022-03/DoDI_6130_06_Use_of_Dietary_Supplements_in_the_DoD_508.pdf

[20] Operation Supplement Safety. Accessed March 4, 2021. https://www.opss.org

[21] Operation Supplement Safety Scorecard. Accessed March 4, 2021. https://www.opss.org/screen-your-supplement-safety-read-label-your-supplement-and-answer-these-questions

[22] Avula B, Chittiboyina AG, Bae JY, . The power of hyphenated chromatography: time of flight mass spectrometry for unequivocal identification of spirostanes in bodybuilding dietary supplements. J Pharm Biomed Anal. 2019;167:74-82. doi:10.1016/j.jpba.2018.12.045 30753977

[23] Crawford C, Boyd C, Avula B, Wang YH, Khan IA, Deuster PA. A public health issue: dietary supplements promoted for brain health and cognitive performance. J Altern Complement Med. 2020;26(4):265-272. doi:10.1089/acm.2019.0447 32119795 PMC7153641

[24] Attipoe S, Manganello C, Scott JM, Deuster PA. Usefulness of a risk assessment tool to risk stratify dietary supplements. Mil Med. 2017;182(11):e2086-e2091. doi:10.7205/MILMED-D-17-00021 29087887

[25] Rittenhouse M, Kegel J, Attipoe S, Deuster P. An innovative dietary supplement scorecard for assessing risk. Nutr Today. 2019;54(6):277-282. doi:10.1097/NT.0000000000000380

[26] US Food and Drug Administration. Label claims for conventional foods and dietary supplements. Accessed March 9, 2021. https://www.fda.gov/food/food-labeling-nutrition/label-claims-conventional-foods-and-dietary-supplements

[27] US Department of Health and Human Services, US Food and Drug Administration. Regulations on statements made for dietary supplements concerning the effect of the product on the structure or function of the body. 21 CFR §101 (2000).

