# Supplementary material for: Analysis of Select Dietary Supplement Products Marketed to Support or Boost the Immune System
Source: JAMA Netw Open. 2022 Aug 10;5(8):e2226040. doi: 10.1001/jamanetworkopen.2022.26040 (PMC9366544; doi:10.1001/jamanetworkopen.2022.26040)
Supplement: Supplement. — eMethods. Analytical Methods eTable. Phytochemicals Reported for the Botanical Plant Parts Used in the Study [file jamanetwopen-e2226040-s001.pdf]

Supplementary Online Content

Crawford C, Avula B, Lindsey AT, et al. Analysis of select dietary supplement products marketed to support or boost the immune system. *JAMA Netw Open*. 2022;5(8):e2226040. doi:10.1001/jamanetworkopen.2022.26040

eMethods. Analytical Methods

eTable. Phytochemicals Reported for the Botanical Plant Parts Used in the Study

This supplementary material has been provided by the authors to give readers additional information about their work.

© 2022 Crawford C et al. *JAMA Network Open.*

eMethods. Analytical Methods

1. Chemicals and reagents

HPLC grade acetonitrile, methanol and formic acid were purchased from Fisher Scientific (Fair Lawn, NJ, USA). Water was obtained using a milliQ-Gradient system. Ascorbic acid, chicoric acid, caftaric acid, adenosine, folic acid, 6-gingerol, rosmarinic acid, berberine, cyanidin 3-*O*-sambubioside-5-*O*-glucoside, isoquercitrin, isorhamnetin 3-*O*-rutinoside, cyanidin 3-*O*-sambubioside, cyanidin 3-*O*-glucoside, cholorogenic acid, rutin, and quercetin were purchased from Sigma (St. Louis, MO, USA).

2. Preparation of dietary supplement samples

The dietary supplements were encountered in the form of either capsules, tablets, powders or liquids. For capsules, 5 items were weighed, opened and their contents were mixed and triturated in a mortar and pestle prior. Each dietary supplement purchased as powders or capsules or tablets, about 1000 mg for powders and average weight in case of capsule content or tablets were weighed into centrifuge tubes, re-suspended with methanol, vortex and sonicated for 30 minutes, following centrifugation for 15 minutes at 959 x g. The procedure was repeated for three times and the clear supernatant was subsequently transferred to a 10 mL volumetric flask. The final volume was adjusted with methanol to 10 mL and mixed thoroughly. Prior to injection, the samples were filtered through a 0.45µm polytetrafluoroethylene (PTFE) membrane filter. For dietary supplements purchased in liquid form, a 1:1 dilution was prepared in methanol, filtered and injected.

3. Instrumentation

Liquid Chromatography-Quadrupole Time-of-Flight Mass Spectrometry (LC-QToF-MS)

The analytical methodology is same as reported elsewhere.[1-3] The liquid chromatographic system is an Agilent Series 1290 and the mass spectrometric analysis was performed with a QToF-MS/MS (Model #G6530A, Agilent Technologies, Palo Alto, CA, USA) equipped with an ESI source with Jet Stream technology. All the operations, acquisition and analysis of data were controlled by Agilent MassHunter Acquisition Software Ver. A.01.00 and operated under MassHunter Workstation software Ver. B.02.00. Each sample was analyzed in both positive and negative modes to provide abundant information for structural identification. Mass spectra are recorded across the range *m/z* = 50-1700 with accurate mass measurement of all mass peaks. MassHunter Workstation software, including Qualitative Analysis (version B.07.00), was used for processing both raw MS and MS-MS data, including molecular feature extraction, background subtraction, data filtering, and molecular formula estimation. The raw data were processed using the Find by Molecular Feature (MF) algorithm called Molecular Feature Extractor (MFE) within MassHunter Qualitative Analysis software. Extracted molecular features were processed to create a list of compounds.

A compound search for the non-targeted compounds were characterized by matching the experimental molecular formula in an [a] The Agilent MassHunter Forensics and Toxicology (>9000 components) Personal Compound Database (PCD) [b] In-House generated library for 11,000 components of medicinal plant samples. Other search engines included SciFinder (web-based version), Dictionary of Natural product (CRC, 2021), and google search engines by molecular formulae were used for the identification of "known unknowns." These approaches have been utilized to identify a wide range of components, including additives, compounds from natural products, etc. In-house library includes the compound name, molecular formula, exact mass, CAS #, and structure of each compound. From the possible positive hits, the results were compared with MS-MS experiments and to those available in literature. All compounds either generated a high-abundance $[M\text{-}H]^-$ or/and $[M+HCOO]^-$ ion in negative mode or a high-abundance $[M+H]^+$ or/and $[M+Na]^+$ ion in positive mode, therefore, the $[M\text{-}H]^-$ or $[M+H]^+$ ions of each compound were selected as the precursor ions for subsequent MS-MS experiments to give more fragment ions. The generation of diagnostic fragment ions provided information concerning the core skeleton and nature of the substituents.

© 2022 Crawford C et al. *JAMA Network Open.*

4. Analysis of Dietary Supplements using LC-QToF

In this study, all dietary supplements were analyzed using LC-QToF method. The operation parameters (fragmentation energies, gas flows and temperatures) of a high-resolution mass spectrometer were designed to deliver high-accuracy qualitative data for the identification of components from botanicals. A full scan MS and MS-MS modes using QToF-MS is effective and sensitive in exploring the identifications of both target and non-target compounds from supplements. The methanolic extracts were subjected to both negative and positive ion modes.

[a] Identification of single component ingredients

The identification of single compound ingredients including water soluble vitamins, quercetin, berberine, melatonin, theanine, biotin, amino acids, beta-hydroxy-beta-methylbutyrate, methylsulfonylmethane, were detected based on the accurate or exact mass and MS-MS spectrum of compounds and in some cases comparing with standard compounds.

Products containing folic acid ($C_{19}H_{19}N_7O_6$ *m/z* 442.147 $[M+H]^+$; $[M-H]^-$ *m/z* 440.1324) was detected in 2 products [#9 and 23] and not detected in one product [#7]. Vitamin B12 ($C_{63}H_{88}CoN_{14}O_{14}P$**,** *m/z* 1355.5747 $[M+H]^+$/678.2913 $[M+2H]^{2+}$) was detected from product # 7 and 23 but was not detected in product # 21. Similar the case with Zinc carnosine ($C_9H_{12}N_4O_3Zn$ *m/z* 289.0274 $[M+H]^+$) or carnosine ($C_9H_{14}N_4O_3$ *m/z* 227.1139 $[M+H]^+$) which was not detected from product # 13.

[b] Identification of botanical ingredients

The botanical raw materials vary greatly in their chemical compositions due to batch variation, cultivar variation, harvesting, processing etc., but the species-specific compounds of any botanical collected at any geographic region will not differ. The content of the compounds will vary but not the species-specific compounds. We usually look for multiple compounds for any botanical ingredient rather than single compound. Well-developed botanical extracts have a characteristic chemical profile or fingerprint that can be used to both determine identity with a high degree of confidence by looking at the suite of compounds. Most of the compound's attribution is done based on the reported papers/literature. If botanical ingredient extracts are added in low amounts (<5 mg), detection might become difficult.

LC-QToF provides the greatest confidence for ensuring the identity with fewer false positives.

For example, elderberry extract (*S. nigra*) fruit detection is attributed to the presence of anthocyanins [cyanidin 3-*O*-sambioside-5-*O*-glucoside (*m/z* 743.2036 $C_{32}H_{39}O_{20}^+$), cyanidin 3-*O*-sambubioside *m/z* 581.1506 $C_{26}H_{29}O_{15}^+$), cyanidin 3-*O*-glucoside *m/z* 449.1084 $C_{21}H_{21}O_{11}^+$)] and phenolic compounds [chlorogenic acid $C_{16}H_{18}O_9$ *m/z* 355.1024, rutin $C_{27}H_{30}O_{16}$ *m/z* 611.1607, isoquercitrin $C_{21}H_{20}O_{12}$ *m/z* 465.1028, isorhamnetin 3-*O*-rutinoside $C_{28}H_{32}O_{16}$ *m/z* 625.1769, quercetin $C_{15}H_{10}O_7$ *m/z* 303.0499].

Overall, 3 products [#5, 18, 22] containing elderberry were adulterated with *Oryza sativa* (black rice), which is based on the detection of peonidin 3-*O*-glucoside and relatively high concentrations of cyanidin 3-*O*-glucoside in these samples. One product [#30] containing *S. nigra* fruit extract, could not detect for its presence.

Another example shown for products [#4 and 14] containing quercetin is also spiked with pure flavonoids including kaempferol, rutin, isoquercetin, isorhamnetin etc or extracts from other flavonoid-rich plant sources.

All other plants not detected in the analyzed dietary supplements are listed in detail with chemical constituents in e**Table**

eReferences

1. Avula B, Chittiboyina AG, Bae JY, et al. The power of hyphenated chromatography-Time of flight mass spectrometry for unequivocal identification of spirostanes in bodybuilding dietary supplements. *J Pharm Biomed Anal.* 2019;167:74-82.
2. Crawford C, Boyd C, Avula B, Wang YH, Khan IA, Deuster PA. A public health issue: Dietary supplements promoted for brain health and cognitive performance. *J Altern Complement Med (New York, NY).* 2020;26(4):265-272.
3. Crawford C, Walter AR, Avula B, et al. Relative safety and quality of various dietary supplement products U.S. Service Members ask about. *Clin Toxicol (Phila).* 2022:1-8.

eTable. Phytochemicals Reported for the Botanical Plant Parts Used in the Study

Product #'s	Common name/ (Scientific name)	Family	Plant Part	Phytochemicals	References
30	Elderberry (*Sambucus nigra*)	Adoxaceae	Fruit	**<u>Anthocyanins:</u>**<br>Cyanidin 3-*O*-sambioside-5-*O*-glucoside, cyanidin 3-*O*-sambubioside, cyanidin 3-*O*-glucoside<br>**<u>Phenolic compounds:</u>**<br>Chlorogenic acid, rutin, isoquercitrin, isorhamnetin 3-*O*-rutinoside, quercetin	[1] [28]
5, 19, 22, 28, 30	Ginger (*Zingiber officinale* L.)	Zingiberaceae	Rhizome	**<u>Phenol compounds:</u>**<br>6-gingerol, 8-gingerol, 10-gingerol, 6-shogaol, 10-shogaol	[2] [3] [28]
19, 28	Oregano (*Origanum vulgare* L.)	Lamiaceae	Leaf	**<u>Phenolic acids and flavonoids:</u>**<br>Sinapic acid, 2-hydroxybenzoic acid, *m*-coumaric acid, rosmarinic acid, 3,7-dimethylquercetin, dihydrobiochanin A, luteolin 7-*O*-glucuronide	[4] [28]
11	Horehound (*Marrubium vulgare*)	Lamiaceae	Aerial parts	**<u>Phenols and flavonoids:</u>**<br>Marrubiin, marruboside, forsynthoside B, samioside, marrubenol, verbascoside, 12-hydroxymarrubiin, apigenin 7-(2-glucosyllactate), luteolin 7-lactate	[5] [28]
12, 19, 28, 30	*Siberian ginseng (Eleutherococcus senticosus)*	Araliaceae	Root	**<u>Eleutherosides, flavonoids and phenolic acids:</u>**<br>Eleutheroside B1, Eleutheroside E2, Eleutheroside D, Eleutheroside A-D, hyperin, rutin, afzelin, quercetin, and kaempferol, gallic, protocatechuic, gentisic, 4-OH-benzoic, 3-OH-benzoic, vanillic, *trans*-caffeic, *cis*-caffeic, syringic, *trans*-*p*-coumaric, *trans*-ferulic, veratric, salicylic, 3-OH-cinnamic, *trans*-sinapic, and *cis*-sinapic acid	[6] [28]
18	Solomon's-Seal (*Polygonatum odoratum*)	Asparagaceae	Rhizome	**<u>Steroidal sapogenins and glycosides/homoisoflavones:</u>**<br>3-*O*-β-D-glucopyranosyl-(1→2)-[β-D-xylo-pyranosyl-(1→3)]-β-D-glucopyranosyl-(1→4)-galactopyranosyl-(25*S*)-spirost-5(6)-en-3β-ol, 3-*O*-β-D-gluco- pyranosyl-(1→2)-[β-D-xylo-pyranosyl-(1→3)]-β-D-glucopyranosyl-(1→4)-galactopyrano-syl-(25*S*)-spirost-5(6),14(15)-dien-3β-ol and 3-*O*-β-D-glucopyranosyl-(1→2)-[β-D-xylopyranosyl-(1→3)]-β-D-glucopyranosyl-(1→4)-galactopyranosyl-(25*S*)-spirost-5(6)-en-3β, 14α-diol, polygodosides A-F, polygodosin A, 5,7-dihydroxy-4′-methoxy-6,8-dimethyl-homoisoflavone, 4′, 5, 7-tri-hydroxy-6,8-dimethylhomoisoflavone, 4′,5,7-trihydroxy-6-methyl-8-dimethoxyhomoisoflavone, 4′,5,7-trihydroxy-6-ethylhomoisoflavone and 5,7-dihydroxy-4′,8-dimethoxy-6-methylhomoisoflavone	[7] [8] [9] [28]
18, 30	Astragalus (*Astragalus membranaceus*)	Fabaceae	Root	**<u>Saponins and flavonoids:</u>**<br>Cycloartane- and oleanane-type saponins including Astramembranosides A/B, Astragaloside I, Astragaloside III, Astragaloside A, Astragaloside IV-VII<br>Calycosin-7-*O*-*β*-D-glucopyranoside, Calycosin, Ononin, Formononetin, Calycosin 7-*O*-*β*-D-{6''-[(*E*)-but-2-enoyl]}-glucoside, Pratensein 7-*O*-*β*-D-glucopyranoside, 6''-Acetylononin, Isomucronulatol 7-*O*-*β*-glucoside, Isomucronulatol, Isoliquiritigenin, Vesticarpan	[10] [28]
10, 17, 18	Woad (*Isatis tinctoria*)	Brassicaceae	Leaf/Root	**<u>Alkaloids, phenolic acids and flavonoids:</u>**<br>**<u>Leaf:</u>** p-hydroxybenzoic, *o*-methoxybenzoic, *p*-methoxybenzoic, dihydrocaffeic, ferulic, sinapic, salicylic, vanillic, and 4-hydroxyphenylacetic acids, Isaindigotone, Indigotilisocoumarin A, Isaindigotidione Vicenin-2, Isoscoparine, Luteolin-6-C-glucoside-7-*O*-glucoside, Luteolin- glucuronide<br>**<u>Root:</u>**<br>Isaindigotone, Isatan A/B, Isatindigoside A, isatindigobisindoloside A	[11] [28]
18	Pau D'Arco	Bigoniaceae	Bark	**<u>Flavonoids/ iridoids/ lignans/phenolic acids:</u>**	[12] [28]

© 2022 Crawford C et al. *JAMA Network Open.*

	(*Tabebuia avellanedae*)			Quercetin, avellaneine A-D, avellanedae A, glochidioboside, 4-*O*-methylcedrusin, veratric acid, 4-hydroxybenzoic acid, 3,4,5-trimethoxybenzoic acid	
18	Goldthread (*Coptis*)	Ranunculaceae	Rhizome	**Protoberberine-type alkaloids:** Berberine, coptisine, jatrorrhizine, palmatine, columbamine, epiberberine, and magnoflorine	[13] [14] [15] [28]
10	Japanese catnip (*Schizonepeta tenuifolia*)	Lamiaceae	Aerial parts	**Terpenes/Phenolics:** (-)-pulegone, piperitenone, schizonepetoside A, schizonepetoside C, (+)-spatulenol, ursolic acid, 2α,3α,24α-trihydroxyolean-12-en-28-oic acid, 5α,8α-epidioxyergosta-6,22-diol-3β-ol, stigmast-4-en-3-one<br>Rosmarinic acid, apigenin-7-*O*-β-D-glucopyranoside, luteolin-7-*O*-β-D-glucuronopyranoside, hesperidin, luteolin, diosmetin	[16] [28]
17	*Polygala tenuifolia*	Polygalaceae	Root	**Saponins, xanthones:** sibiricose A5, sibiricose A6, glomeratose A, tenuifoliside A, tenuifoliside B, tenuifoliside C, sibiricaxanthone B, and polygalaxanthone III	[17] [28]
28	Garlic (*Allium sativum* L.)	Amaryllidaceae	Bulb	**Polyphenols and organosulfur compounds:** γ-glutamyl-S-alk(en)yl-L-cysteines and S-alk(en)yl-L-cysteine sulfoxides, allicin and deoxyalliin and particularly L-alliin as the major sulfur-containing compound	[18] [19] [20] [21] [28]
17	Licorice (*Glycyrrhiza uralensis*, *G. glabra*, *G. inflata*)	Fabaceae	Root	**Flavonoids and Triterpene Saponins:** **Species-specific markers** including glabridin, glycybridins, hispaglabridins, glabrol from *G. glabra*, glycycoumarin, licoflavonol, licoisoflavone A/B from *G. uralensis*, and licochalcones A-E from *G. inflata* were identified. Liquiritin apioside, ioliquiritin, liquiritigenin, naringenin, glycyrrhizin, formononetin were detected in all species of Glycyrrhiza	[22] [28]
13	Slippery elm (*Ulmus rubra* Muhl)	Ulmaceae	Inner Bark	**Triterpenes:** Oleanolic acid, ursolic acid, uvaol, betulinic acid	[23] [24]
13	*Aloe vera*	Asphodelaceae	Leaf	**Anthraquinones:** Aloe-emodin, Emodin, Aloin A/B	[25] [26] [28]
30	Tasmanian blue gum (*Eucalyptus globulus*)	Myrtaceae	Leaf	**Terpenoids, tannins, flavonoids and phloroglucinol derivatives:** gallic acid, eucaglobulin, globulusin B, globulol, euglobal Ia1, euglobal IIb, euglobal Ic, euglobal IIc, euglobal Ia2, euglobal IIa, euglobal Ib, 2,4-diformylphloroglucinol, quercetin 3-*O*-glycoside, quercetin 3-*O*-rhamnoside, quercetin 3-*O*-β-D-glucuronide.	[27] [28]
30	Goldenseal (*Hydrastis canadensis* L.)	Ranunculaceae	Root	**Isoquinoline alkaloids:** β-hydrastine, hydrastine, berberine, berberastine, canadine	[28] [29]

*These plants described in the eTable 1 claimed in some of the dietary supplements were not detected and the product codes of these supplements are listed in column 1 of this table to align with products listed in Table 1 of the main article.

References to eTable 1:

[1] Avula B, Katragunta K, Wang Y-H, et al. Chemical profiling and UHPLC-QToF analysis for the simultaneous determination of anthocyanins and flavonoids in *Sambucus* berries and authentication and detection of adulteration in elderberry dietary supplements using UHPLC-PDA-MS. J Food Compost Anal. 2022; 110 (104584) ISSN 0889-1575, https://doi.org/10.1016/j.jfca.2022.104584.

[2] Schwertner HA, Rios DC. High-performance liquid chromatographic analysis of 6-gingerol, 8-gingerol, 10-gingerol, and 6-shogaol in ginger-containing dietary supplements, spices, teas, and beverages, *J Chromatogr B Analyt Technol Biomed Life Sci.* 2007; 856(1–2): 41-47. ISSN 1570-0232, https://doi.org/10.1016/j.jchromb.2007.05.011.

[3] Tao Y, Li W, Liang W, Van Breemen RB. Identification and quantification of gingerols and related compounds in ginger dietary supplements using high-performance liquid chromatography-tandem mass spectrometry. *J Agric Food Chem*. 2009;57(21):10014-10021. doi:10.1021/jf9020224

© 2022 Crawford C et al. *JAMA Network Open.*

[4] Ali A, Bashmil YM, Cottrell JJ, Suleria HAR, Dunshea FR. LC-MS/MS-QTOF screening and identification of phenolic compounds from australian grown herbs and their antioxidant potential. *Antioxidants (Basel).* 2021;10(11):1770. doi: 10.3390/antiox10111770.

[5] Amri B, Martino E, Vitulo F, et al. *Marrubium vulgare* L. leave extract: phytochemical composition, antioxidant and wound healing properties. *Molecules.* 2017; 22(11):1851. doi: 10.3390/molecules22111851.

[6] Załuski D, Olech M, Galanty A, et al. Phytochemical content and pharma-nutrition study on *Eleutherococcus senticosus* fruits intractum. *Oxid Med Cell Longev.* 2016; 9270691. doi: 10.1155/2016/9270691.

[7] Wang DM, Li DW, Zhu W, Zhang JF, Peng P. Steroidal saponins from the rhizomes of *Polygonatum odoratum*. *Natural Product Research. 2009;* 23, 940-947.

[8] Zhao X, Li J. Chemical constituents of the genus *Polygonatum* and their role in medicinal treatment. *Nat Prod Commun.* 2015;10(4):683-8.

[9] Zhang H, Chen L, Kou JP, Zhu DN, Qi J, Yu BY. Steroidal sapogenins and glycosides from the fibrous roots of Polygonatum odoratum with inhibitory effect on tissue factor (TF) procoagulant activity. *Steroids.* 2014 Nov; 89:1-10. doi: 10.1016/j.steroids.2014.07.002.

[10] Li X, Qu L, Dong Y, et al. A review of recent research progress on the astragalus genus. *Molecules.* 2014; 19(11):18850-80. doi: 10.3390/molecules191118850.

[11] Speranza J, Miceli N, Taviano MF, et al. *Isatis tinctoria* L. (Woad): A review of its botany, ethnobotanical uses, phytochemistry, biological activities, and biotechnological studies. *Plants (Basel)*. 2020;9(3):298. doi:10.3390/plants9030298

[12] Ashraf Nageeb Elsayed Hamed, Basma Khalaf Mahmoud, Mamdouh Nabil Samy, Mohamed Salah Kamel. An extensive review on genus "Tabebuia", family bignoniaceae: Phytochemistry and biological activities (1967 to 2018). *Journal of Herbal Medicine*. 2020; 24, 100410, ISSN 2210-8033, https://doi.org/10.1016/j.hermed.2020.100410.

[13] Liu Y, Wang B, Shu S, et al. Analysis of the *Coptis chinensis* genome reveals the diversification of protoberberine-type alkaloids. *Nat Commun.* 2021; 12, 3276. https://doi.org/10.1038/s41467-021-23611-0

[14] Lv X, Li Y, Tang C, Zhang Y, Zhang J, Fan G. Integration of HPLC-based fingerprint and quantitative analyses for differentiating botanical species and geographical growing origins of Rhizoma coptidis. *Pharm. Biol.* 2016; 54, 3264–3271.

[15] Yang Y, Peng J, Li F, Liu X, Deng M, Wu H. Determination of alkaloid contents in various tissues of *Coptis chinensis* Franch by reversed phase-high performance liquid chromatography and ultraviolet spectrophotometry. *J Chromatogr Sci*. 2017; 55, 556–563.

[16] Lee IK., Kim Lee, SY, Hong JK, Lee, J*H. Phytochemical Constituents of Schizonepeta tenuifolia Briquet. Natural Product Sciences. 2008; 14, 100-106.*

[17] Xu R, Mao F, Zhao Y, et al. UPLC Quantitative analysis of multi-components by single marker and quality evaluation of *Polygala tenuifolia* wild extracts. *Molecules*. 2017;22(12):2276. doi:10.3390/molecules22122276

[18] El-Saber Batiha G, Magdy Beshbishy A, G Wasef L, et al. Chemical constituents and pharmacological activities of garlic (*Allium sativum* L.): A Review. *Nutrients*. 2020;12(3):872. doi: 10.3390/nu12030872.

[19] Lanzotti, V. The analysis of onion and garlic. *J Chromatography A.* 2006; 1112(1–2): 3-22. ISSN 0021-9673, https://doi.org/10.1016/j.chroma.2005.12.016.

[20] I Arnault, J.P Christidès, N Mandon, T Haffner, R Kahane, J Auger, High-performance ion-pair chromatography method for simultaneous analysis of alliin, deoxyalliin, allicin and dipeptide precursors in garlic products using multiple mass spectrometry and UV detection, *J Chromatography A.* 2003; 991(1):69-75. ISSN 0021-9673, https://doi.org/10.1016/S0021-9673(03)00214-0.

[21] Amagase H, Petesch BL, Matsuura H, Kasuga S, Itakura Y. Intake of garlic and its bioactive components. *J Nutr*. 2001; 131, 955S–962S

[22] He M, Lv HY, Li YP, et al. A multiplex approach for the UPLC-PDA-MS/MS data: analysis of licorice. *Anal Methods.* 2014; 6(7), 2239-2246. https://doi.org/ 10.1039/C3AY41861H

[23] Park WS, Kim HJ, Khalil AAK, et al. Anatomical and chemical characterization of *Ulmus* Species from South Korea. *Plants (Basel)*. 2021; 10(12):2617. doi: 10.3390/plants10122617.

[24] Wijesundara NM, Sekhon-Loodu S, Rupasinghe HV. Phytochemical-rich medicinal plant extracts suppress bacterial antigens-induced inflammation in human tonsil epithelial cells. *PeerJ.* 2017;5:e3469. doi: 10.7717/peerj.3469.

[25] Wang G, Su P, Zhang F, et al. Comparison of microwave-assisted extraction of aloe-emodin in aloe with Soxhlet extraction and ultrasound-assisted extraction. *Sci China Chem.* 2011; 54, 231–236. https://doi.org/10.1007/s11426-010-4017-9

© 2022 Crawford C et al. *JAMA Network Open.*

[26] Guo X, Mei N. Aloe vera: A review of toxicity and adverse clinical effects. *J Environ Sci Health C Environ Carcinog Ecotoxicol Rev.* 2016; 34(2):77-96. doi: 10.1080/10590501.2016.1166826.

[27] Boulekbache-Makhlouf L, Meudec E, Mazauric JP, Madani K, Cheynier V. Qualitative and semi-quantitative analysis of phenolics in Eucalyptus globulus leaves by high-performance liquid chromatography coupled with diode array detection and electrospray ionisation mass spectrometry. *Phytochem Anal.* 2013;24(2):162-70. doi: 10.1002/pca.2396.

[28] Dictionary of natural products v30.2, CRC Press, Taylor & Francis group 2021.

[29] Avula B, Wang YH, Khan IA. Quantitative determination of alkaloids from roots of *Hydrastis canadensis* L. and dietary supplements using Ultra-Performance Liquid Chromatography with UV detection. *J AOAC INTERNATIONAL*. 2012;95(5): 1398–1405. https://doi.org/10.5740/jaoacint.12-0

© 2022 Crawford C et al. *JAMA Network Open.*
